# Physical Activity Levels in Peripheral Artery Disease Patients

**DOI:** 10.5935/abc.20190142

**Published:** 2019-09

**Authors:** Aline Mendes Gerage, Marilia de Almeida Correia, Paulo Mesquita Longano de Oliveira, Aline Cabral Palmeira, Wagner Jorge Ribeiro Domingues, Antônio Eduardo Zeratti, Pedro Puech-Leão, Nelson Wolosker, Raphael Mendes Ritti-Dias, Gabriel Grizzo Cucato

**Affiliations:** 1 Universidade Federal de Santa Catarina - Departamento de Educação Física, Florianópolis, SC - Brazil; 2 Universidade Nove de Julho, São Paulo, SP - Brazil; 3 Hospital Israelita Albert Einstein, São Paulo, SP - Brazil; 4 Universidade Federal do Amazonas, Parintins, AM - Brazil; 5 Universidade de São Paulo Faculdade de Medicina Hospital das Clinicas, São Paulo, SP - Brazil

**Keywords:** Motor Activity, Exercise, Waling, Peripheral Arterial Disease, Intermittent Claudication

## Abstract

**Background:**

Increases in daily physical activity levels is recommended for patients with peripheral artery disease (PAD). However, despite this recommendation, little is known about the physical activity patterns of PAD patients.

**Objective:**

To describe the physical activity patterns of patients with symptomatic peripheral artery (PAD) disease.

**Methods:**

This cross-sectional study included 174 PAD patients with intermittent claudication symptoms. Patients were submitted to clinical, hemodynamic and functional evaluations. Physical activity was objectively measured by an accelerometer, and the time spent in sedentary, low-light, high-light and moderate-vigorous physical activities (MVPA) were obtained. Descriptive analysis was performed to summarize patient data and binary logistic regression was used to test the crude and adjusted associations between adherence to physical activity recommendation and sociodemographic and clinical factors. For all the statistical analyses, significance was accepted at p < 0.05.

**Results:**

Patients spent in average of 640 ± 121 min/day, 269 ± 94 min/day, 36 ± 27 min/day and 15 ± 16 min/day in sedentary, low-light, high-light and MVPA, respectively. The prevalence of patients who achieved physical activity recommendations was 3.4%. After adjustment for confounders, a significant inverse association was observed between adherence to physical activity recommendation and age (OR = 0.925; p = 0.004), while time of disease, ankle brachial index and total walking distance were not associated with this adherence criteria (p > 0.05).

**Conclusion:**

The patterns of physical activity of PAD patients are characterized by a large amount of time spent in sedentary behaviors and a low engagement in MVPA. Younger patients, regardless of the clinical and functional factors, were more likely to meet the current physical activity recommendations.

## Introduction

Patients with peripheral artery disease (PAD) and symptoms of intermittent claudication have walking impairment, several comorbid conditions and increased cardiovascular risk,^1,2^ due to the disease characteristics and severity. Supervised exercise training has been considered a cornerstone in the clinical therapeutic approach in PAD patients,^3^ as it improves several components of physical function and quality of life.^4-6^ Similarly, positive effects of device-monitored, home-based exercise training programs to improve the walking capacity in these patients have also been reported.^7^ However, these interventions are available for a restricted number of patients, limiting applicability in the public health context. Therefore, recommendations to increase physical activity levels remain the most often used approach in clinical practice.

Current physical activity recommendations for the overall population, including PAD patients, consists of practicing at least 150 min of moderate or 75 min of vigorous physical activities or an equivalent combination of moderate-vigorous physical activities (MVPA) per week.^8^ Furthermore, it has been recommended that MVPA should be performed in bouts with at least a 10-minute duration.^8^ Surprisingly, there are no data indicating the number of symptomatic PAD patients who achieve these physical activity recommendations. Given that most of symptomatic PAD patients are older, have several comorbidities, and that symptoms of intermittent claudication are the main barrier for physical activity practice in these patients,^9^ by limiting their walking and functional capacity, it is expected that only a small percentage of the patients would achieve the recommended physical activity levels.

Thus, in this study we aimed to describe the physical activity pattern of Brazilian patients with PAD and symptoms of intermittent claudication according to the recommendations for physical activity practice, providing objective information regarding the time spent in sedentary behavior, light physical activity and MVPA. Moreover, we tested the association between adherence to physical activity recommendations and sociodemographic and clinical factors in Brazilian patients with symptomatic PAD.

## Methods

### Study design and ethical issues

This descriptive study was approved by Local Ethics Committee. Prior to data collection, patients were informed about the methodological and logistic procedures required to participate in the study, as well as the risks and benefits, and signed a written informed consent form before participation.

### Participants

The overall sample consisted of symptomatic PAD patients, recruited at a tertiary center specialized in vascular disease, between September 2015 and November 2017. The tertiary center is a specific unit designed to treat PAD patients with intermittent claudication symptoms. There, physicians instruct patients to: stop smoking, control their risk factors, and increase their physical activity levels. In the present study, no additional instructions were given, and patients were asked to keep their physical activity routine. To be included in the present study, patients should: have PAD (Fontaine Stage II), ankle brachial index (ABI) <0.90 in one or both legs and undergo the six-minute walking test (6MWT). Patients with non-compressible vessels, amputated limbs and/or ulcers, previous diagnosis of neurological or psychiatric disorders, or those classified as illiterate were excluded.

### Measurements

#### Clinical data

A standardized face-to-face interview was performed, including assessment of social and demographic information, co-morbid conditions (self-reported), and medications. Social and demographic variables included age and gender (male or female). Time of disease diagnosis was obtained through the question “*How long have you had the disease?*”. Data on smoking habits (ex- or current smoker, or non-smoker), obesity (body mass index (BMI) ≥ 30 kg/m^2^), diabetes (doctor-diagnosed or hypoglycemic drugs), hypertension (systolic/diastolic blood pressure >140/90 mmHg or antihypertensive drug use), dyslipidemia (doctor-diagnosed or hypolipidemic drug use), coronary heart disease, heart failure and history of cancer (self-reported or analysis of medical records) were obtained.

#### Disease severity

PAD severity was obtained by calculating the ABI in accordance with the guidelines.^10^ All measures were carried out by a single and trained evaluator, using vascular Doppler (Medmega DV160, Brazil) and aneroid sphygmomanometer.

#### Walking capacity

The 6MWT was performed on a 30-meter long corridor, following the previously described protocol.^11^ Briefly, patients were instructed to complete as many laps as possible. Patients were encouraged to “walk at the usual pace for six-minutes and cover as much ground as possible”. Patients were informed that they could rest, if necessary. At the end of each minute, patients received feedback on the elapsed time and standardized encouragement in the form of statements such as “you are doing well, keep it up” and “do your best". Total walking distance was defined as the maximum distance which the patient could walk during the test, with or without leg pain. In addition, the self-reported ambulatory ability was assessed using the Brazilian versions of Walking Impairment Questionnaire (WIQ)^12^ and the Walking Estimated-Limitation Calculated by History (WELCH) questionnaire.^13^

#### Objectively measured physical activity

Physical activity was assessed using a GT3X+ triaxial accelerometer (Actigraph, Pensacola, FL, USA). Each participant was instructed to use the accelerometer for seven consecutive days, removing it only for sleeping, bathing or performing activities in the water. The device was attached to an elastic belt and attached to the right side of the hip. Data reduction was performed using the Actilife software, version 6.02 (Actigraph, Pensacola, FL, USA), with a 60Hz sample frequency and 60s epochs. Periods with consecutive values of zero for 60 min or longer were interpreted as “accelerometer not worn” and excluded from the analysis. Physical activity data were included only if the participant had accumulated a minimum of 10 hours/day of recording for at least four days, including one weekend day. The average of total time spent in each intensity of physical activity was calculated using the cutoff points specific for elderly individuals,^14^ adapted by Buman et al.,^15^ considering sedentary time (SED) as 0 - 99 counts/min; low-light physical activities as 100-1040 counts/min, high-light physical activities as 1041-1951 counts/min and MVPA as ≥ 1952 counts/min using the vertical axis, and analyzed in min/day, adjusting for the time and number of days the device was worn. The total time spent in SED bouts and the time spent in bouts of at least high-light physical activities and MVPA were analyzed by the sum of minutes spent in SED, high-light physical activities and MVPA, respectively, in periods lasting ≥10 minutes. Additionally, we calculated the percentage of patients that met the current physical activity recommendations (≥ 150 min/week) considering MVPA bouts.

### Statistical analysis

The sample size was calculated by estimating an effect size of 0.3 in the chi-square analysis, considering an alpha error of 5% and a power of 80%. The sample size required for the study was 143 participants. The data were stored and analyzed using the Statistical Package for the Social Sciences (SPSS, version 17.0, SPSS Inc, Chicago, IL). Descriptive analysis was performed to summarize the patients’ data using means, standard deviation or frequency distribution (absolute and relative), as appropriate. Binary logistic regression was used to test the crude and adjusted (age, time of the disease diagnosis, ankle-brachial index, and six-minute walking distance) association between adherence to physical activity recommendation and sociodemographic data and clinical factors. The results are expressed as odds ratios (OR) and their respective 95% confidence intervals (95%CI). For all the statistical analyses, significance was set at p < 0.05.

## Results

The overall characteristics of patients are shown in Table 1. The mean age of all patients was 66.7 ± 9.0 years and, on average, patients had moderate disease (ABI: 0.61 ± 0.18). Most patients had hypertension (88.9%), dyslipidemia (85.2%) and diabetes (52.4%),and used antihypertensive (78%) (i.e. thiazide diuretics, calcium channel blockers, angiotensin-converting enzyme inhibitors, angiotensin II receptor antagonists, beta-blockers), lipid-lowering (89%) (i.e. statins) and antiplatelet agent drugs (85%) (i.e. irreversible cyclooxygenase inhibitors, adenosine diphosphate receptor inhibitors). Forty-three percent of the patients used antidiabetic (i.e. sulfonylureas, metformin, thiazolidinediones, alpha-glucosidase inhibitors, meglitinides), 29% used vasodilator (i.e. hydralazine and minoxidil) and 20% used antidepressant drugs (i.e. sertraline, fluoxetine, citalopram, escitalopram, paroxetine).

**Table 1:** Characteristics of peripheral artery disease patients according to gender (n = 174)

	Values
Age (years)	66.7 (9.0)
Gender (% men)	61.5
Still working (%)	20.5
Time of disease diagnosis (yrs.)	7.9 (5.8)
Ankle-brachial index	0.61 (0.18)
Claudication distance (m)^†^	135.9 (82.4)
Six-minute walking distance (m)	326.6 (92.7)
WIQ distance (score)	22.7 (22.2)
WIQ speed (score)	23.2 (15.6)
WIQ stairs (score)	30.7 (25.3)
WELCH (score)	27.3 (19.1)
**Comorbidities and risk factors**	
Charlson index (score)	3.0 (1.7)
Current smokers (%)	18.1
Hypertension (%)	88.9
Dyslipidemia (%)	85.2
Diabetes (%)	52.4
Obesity (%)	28.6
Coronary artery disease (%)	34.5
Heart failure (%)	13.6
Cancer (%)	14.9
**Medications**	
Antihypertensive (%)	78
Antidiabetic (%)	43
Vasodilator (%)	29
Lipid-lowering (%)	89
Antiplatelet agent (%)	85
Antidepressants (%)	20
**Medications**	
Cardiac (%)	24
Vascular (%)	12

WIQ: Walking Impairment Questionnaire; WELCH: Walking Estimated-Limitation Calculated by History.

**Figure 1:**
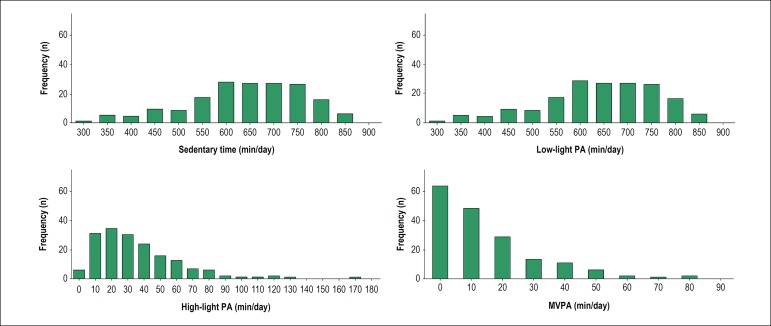
Time spent in sedentary, low light, high light and moderate-to-vigorous (MVPA) physical activities (PA).

Figure 1 depicts the distribution of time spent in sedentary, low-light, high-light and moderate/vigorous activities. Patients, aged between 43 and 96 years, spent in average 640 ± 121 min/day, 269 ± 94 min/day, 36 ± 27 min/day and 15 ± 16 min/day in sedentary, low-light, high-light and moderate/vigorous physical activities, respectively. Most patients (52.9%) spent less than 10 min in moderate/vigorous physical activities (sporadic, non-bouted) per day.

Table 2 depicts data about sedentary bouts (< 100 counts), bouts of high light and MVPA (≥ 1041 counts) and bouts only of MVPA (≥ 1952 counts). Ninety percent of patients spent at least 10 bouts in sedentary behavior per day and, on average, the total duration of this bout was 413.7 ± 151.1 min/day. On the other hand, sedentary breaks lasted 174.4 ± 51.4 min/day. Thirty-one percent of patients did not accumulate 10 or more consecutive minutes a week, at least, in high-light physical activities. Considering only MVPA, 67.7% of patients did not accumulate 10 consecutive minutes (bouts) or more at this intensity of physical activity during a week. Among those patients who spent at least one bout of MVPA, the duration of this bout was 9.7 ± 9.6 min/day.

**Table 2:** Total time spent in sedentary, high light or MVPA and MVPA bouts and sedentary breaks per week and per day in PAD patients (n = 174)

Variable	In a week (mean ± SD)	In a day (mean ± SD)
Total SED bouts	120.1 ± 32.6	17.2 ± 4.7
Total time in SED bouts (min)	2895.6 ± 1057.3	413.7 ± 151.1
Total SED breaks	118.7 ± 32.6	17.0 ± 4.7
Total time in SED breaks (min)	8543.9 ± 2518.0	174.4 ± 51.4
Total high light and MVPA bouts	5.7 ± 7.8	0.8 ± 1.1
Total time in high light and MVPA bouts (min)	84.01 ± 123.8	12.1 ± 17.7
Total MVPA bouts	1.5 ± 3.1	0.22 ± 0.44
Total time in MVPA (min)	22.7 ± 50.3	3.2 ± 7.2

SED: sedentary; MVPA: moderate/vigorous physical activity.

**Figure 2:**
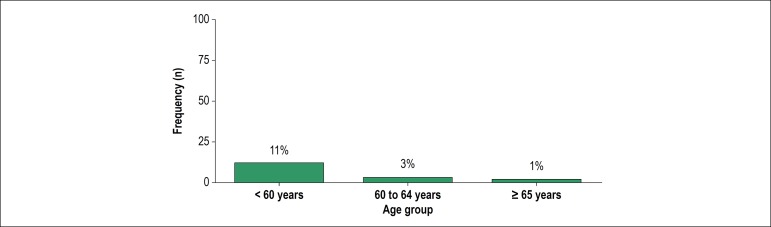
Frequency of PAD patients who achieved the current physical activity recommendations according to age group.

The prevalence of patients who achieved physical activity recommendations for the overall population (≥ 150 min/week of MVPA in bouts of 10 minutes or more) was only 3.4%. Stratifying by age (Figure 2), this prevalence was 11.1% in those under 60 years old, 2.9% in those between 60 and 64 years old, and 1% in those over 65 years. No patients over 70 years old achieved the physical activity recommendations for the overall population.

Table 3 shows crude and adjusted association between adherence to physical activity recommendations and sociodemographic and clinical characteristics in PAD patients. After adjustment for confounders, an inverse and significant association was observed between adherence to physical activity recommendation and age (OR = 0.867; p = 0.011), which means that for each year of life, the odds are ~13% less to meet the physical activity recommendations. Time of disease diagnosis, ABI and total walking distance were not associated with this adherence criterion (p > 0.05).

**Table 3:** Crude and adjusted association between adherence to physical activity recommendations and sociodemographic or clinical characteristics in PAD patients (n = 174)

Variable	Crude analysis		Adjusted analysis*	
	OR (95%CI)	p	OR (95%CI)	p
Age	0.87 (0.79; 0.97)	0.01	0.88 (0.80; 0.98)	0.02
Time of disease diagnosis	0.94 (0.78; 1.12)	0.46	0.98 (0.83; 1.16)	0.82
Ankle-brachial index	0.19 (0.02; 153.41)	0.77	1.14 (0.07; 173.68)	0.96
Six-minute walking distance	1.01 (0.99; 1.02)	0.12	1.00 (0.99; 1.02)	0.32

* Adjusted by age, time of the disease diagnosis, ankle brachial index, and six-minute walking distance.

## Discussion

The main findings of the present study were: a) Brazilian PAD patients with intermittent claudication symptoms spent most part of the day in sedentary behaviors with a short time in MVPA; b) only 3.4% of the patients met the physical activity recommendations for the overall population; c) younger patients, regardless of clinical or physical factors, were more likely to meet the current physical activity recommendations for the overall population.

The cutoff used in the present study considered, in addition to “sedentary” and “moderate-to-vigorous physical activity”, the “low-light” and “high-light” categories.^15^ This decision was based on the following aspects: a) light physical activities are the physical activities most often performed by the elderly, especially those with functional capacity limitations (i.e. patients with PAD); b) light physical activity was broadly unspecified to account for all activity between sedentary and moderate-to-vigorous physical activity (100-1,951 counts/minute); c) the association between light physical activity and health parameters increases when those light physical activities with high energy expenditure (high-light physical activity), that are closer to the classification of moderate-to-vigorous physical activities than sedentary activities, are considered.^15^

In the present study, our sample of PAD patients with intermittent claudication symptoms spent 640 min/day and 15 min/day in sedentary behavior and MVPA, respectively, which represents 66.7% and 1.5% of the waking hours of the day. This pattern is similar to that observed in patients with other cardiovascular diseases, including coronary heart disease, congestive heart failure, myocardial infarction^16^ and stroke survivors.^17^ In these populations, sedentary behavior ranged from 576 min/day^16^ to 606 min/day,^16,17^ while MVPA ranged from 8.6 min/day to 11.4 min/day. Interestingly, although pain symptoms (intermittent claudication) during exercise have been reported as a main barrier for physical activity practice in PAD patients,^9^ their physical activity patterns seem to be similar to cardiac patients without walking impairment. The current physical activity recommendation for the overall population includes 150 min/day of MVPA in bouts of at least 10 min. The results of this study indicated that a very small percentage (3.4%) of our sample met the current physical activity recommendations. These values are lower than those of previous studies generally carried out with adults (~10%),^18^ older adults (12%)^19^ and osteoarthritis patients (13% men and 8% women)^20^ who usually also have physical limitations. The reduced number of patients who met the physical activity recommendations could be explained by the difficulty of PAD patients to perform moderate and/or vigorous physical activities. In fact, as higher-intensity physical activities may precipitate the occurrence of intermittent claudication symptoms, PAD patients commonly perform lower-intensity physical activities to avoid the symptoms.

In the present study, we also analyzed the frequency of patients who achieved the current physical activity recommendations according to age group. We observed that no patients over 70 years old met the current physical activity recommendations for the overall population. This result was confirmed by the multivariate analysis, which revealed that younger patients are more likely to achieve the current physical activity recommendations. These results are in accordance with previous studies carried out with a representative sample of adults from the United States^21^ and with older adults in a population-based sample from Brazil,^19^ which showed an inverse relationship between age and the amount of time spent in MVPA physical activities. The decrease in physical activity with increasing age might be due to a worsening in physical functions associated with the presence of the comorbid conditions, leading to an increase in sedentary behavior and functional capacity impairment.

The ABI, considered one of the best prognostic indexes in PAD,^22^ and walking capacity, a main clinical marker of PAD associated with endothelial function^23^ inflammation^24^ and several clinical indicators,^2,25^ were not associated with the meeting of physical activity recommendations. These results are not surprising, since ABI^26^ and walking capacity have been poorly associated with physical activity in patients with PAD.^27^

Previous studies showed that low levels of physical activity and high levels of sedentary behavior were associated with several risk factors, such as high blood pressure,^28^ increased arterial stiffness,^29^ increased waist circumference and reduced HDL cholesterol,^30,31^ in healthy and clinical populations. In symptomatic PAD patients, a study carried out by Garg et al.^32^ reported that reduced physical activity was associated with increased mortality and cardiovascular events. In other words, patients who attempted to control or eliminate their intermittent claudication symptoms by reducing their physical activity, worsened their risk of myocardial infarction, stroke, and death. Thus, the finding of our study that the majority of PAD patients did not attain the current physical activity recommendations highlights the necessity of interventions to increase physical activity in these patients. Future studies are necessary to describe whether different forms of exercise, home-based programs or wearable physical activity monitors are more effective to help patients to attain the current physical activity recommendations.

The present study has several limitations. Although the accelerometer has been considered a gold standard method to measure physical activities in free-living conditions, it was not possible to measure the type and the context in which the physical activity was performed, which hinders the analysis of what kind of activities were most often performed by these patients. In addition, the accelerometer does not assess physical activities such as water gymnastics and resistance training, which are commonly performed by elderly patients, and could underestimate the real physical activity levels of our sample. Given that there are no specific physical activity recommendation for PAD patients, we employed the current physical activity recommendations for the overall population. However, whether this approach is ideal for PAD patients is unknown. The study was performed in São Paulo, Brazil, and our results may not be extrapolated to other patients with different cultures and lifestyle. We did not include a matched overall population group to compare the prevalence of physical activity between non-PAD and PAD patients. Finally, we did not analyze the type of physical activity performed by these patients, or the difference in physical activities over the year. Some patients assessed during colder/rainier months could be less active than those assessed in the summer months.

## Conclusion

This study showed that the pattern of physical activity of Brazilian PAD patients with intermittent claudication symptoms are characterized by a high amount of time spent in sedentary behavior and a low engagement in MVPA, with only 3.4% of these patients meeting the current physical activity recommendations for the overall population. Moreover, younger patients, regardless of clinical and functional factors, are more likely to meet the current physical activity recommendations.
